# Supplementary material for: The Acute and Short-Term Inhalation of Carbon Nanofiber in Sprague-Dawley Rats
Source: Biomolecules. 2022 Sep 22;12(10):1351. doi: 10.3390/biom12101351 (PMC9599497; doi:10.3390/biom12101351)
Supplement: Supplementary file 1 [file biomolecules-12-01351-s001.zip › biomolecules-1849316-supplementary.pdf]

Supplements

Acute and short-term inhalation study on Carbon nanofiber in Sprague-Dawley Rats

Figure S1. CNF manufacturing methods

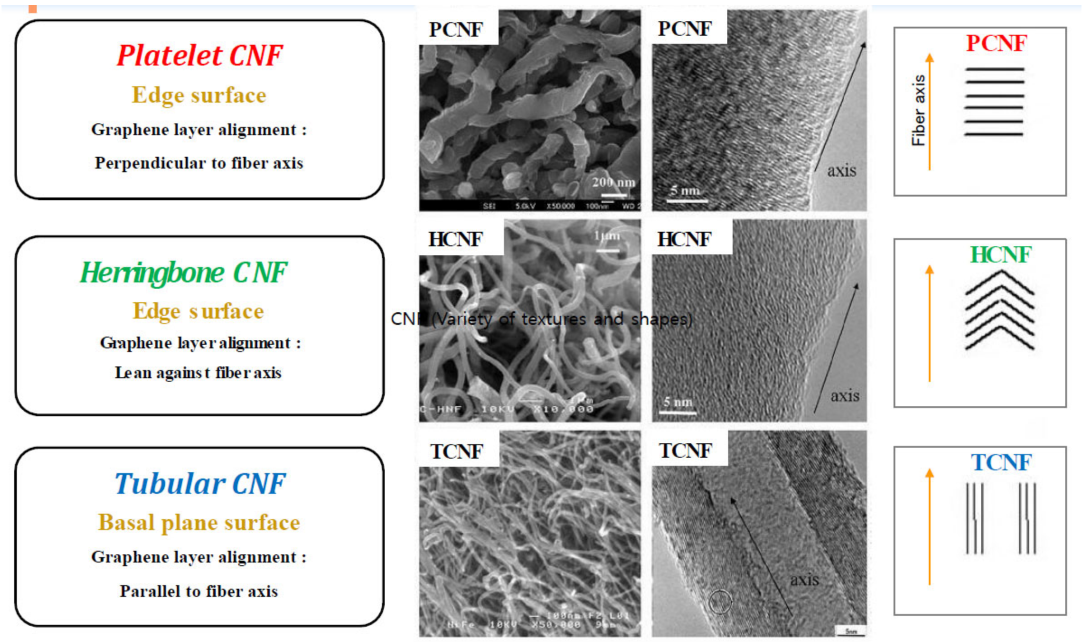

Figure S2. Inhalation chamber system

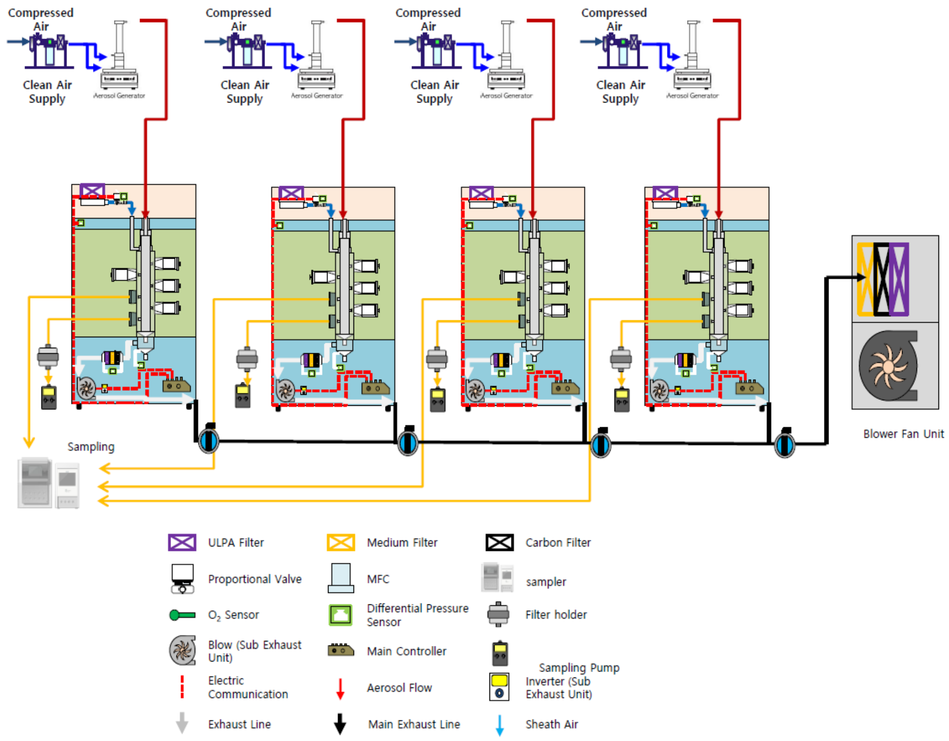

Table S1. Relative organs weights of male rats at 1 day after CNF exposure

Summary of Relative Organ Weights				
(mean ± S.E)	UNIT : g			SEX: MALE
Group:	Control	Low	Moderate	High
BODY WEIGHT	230.94 ± 1.01 (5)	226.38 ± 1.86 (5)	223.22 ± 7.99 (5)	220.98 ± 3.16 (5)
BRAIN	0.84 ± 0.01 (5)	0.84 ± 0.01 (5)	0.87 ± 0.03 (5)	0.86 ± 0.01 (5)
EYE (LEFT)	0.13 ± 0.00 (5)	0.13 ± 0.00 (5)	0.13 ± 0.00 (5)	0.14 ± 0.01 (5)
EYE (RIGHT)	0.13 ± 0.01 (5)	0.13 ± 0.01 (5)	0.12 ± 0.00 (5)	0.14 ± 0.01 (5)
SPLEEN	0.24 ± 0.01 (5)	0.22 ± 0.01 (5)	0.23 ± 0.02 (5)	0.23 ± 0.01 (5)
KIDNEY (LEFT)	0.44 ± 0.01 (5)	0.41 ± 0.01 (5)	0.42 ± 0.01 (5)	0.42 ± 0.01 (5)
KIDNEY (RIGHT)	0.44 ± 0.02 (5)	0.41 ± 0.02 (5)	0.43 ± 0.01 (5)	0.42 ± 0.01 (5)
THYMUS	0.24 ± 0.01 (5)	0.26 ± 0.01 (5)	0.25 ± 0.02 (5)	0.28 ± 0.02 (5)
TESTIS (LEFT)	0.56 ± 0.03 (5)	0.58 ± 0.02 (5)	0.53 ± 0.02 (5)	0.59 ± 0.02 (5)
TESTIS (RIGHT)	0.56 ± 0.03 (5)	0.57 ± 0.01 (5)	0.55 ± 0.03 (5)	0.56 ± 0.03 (5)
HEART	0.42 ± 0.02 (5)	0.45 ± 0.02 (5)	0.44 ± 0.01 (5)	0.48 ± 0.04 (5)
LIVER	3.09 ± 0.06 (5)	2.99 ± 0.04 (5)	3.06 ± 0.09 (5)	2.24 ± 0.63 (5)
LUNG (LEFT)	0.15 ± 0.00 (5)	0.16 ± 0.00 (5)	0.14 ± 0.01 (5)	0.16 ± 0.01 (5)
LUNG (RIGHT)	0.11 ± 0.01 (5)	0.11 ± 0.00 (5)	0.12 ± 0.00 (5)	0.12 ± 0.00 (5)
BRONCHUS	0.04 ± 0.00 (5)	0.05 ± 0.00 (5)	0.05 ± 0.01 (5)	0.03 ± 0.00 (5)

( ): number of animals

Table S2. Relative organs weights of male rats at 3 days after CNF exposure

Summary of Relative Organ Weights																
(mean ± S.E)					UNIT: g											SEX: MALE
Group:	Control				Low				Moderate				High			
BODY WEIGHT	268.58	±	6.33	(5)	271.86	±	6.51	(5)	264.92	±	7.22	(5)	263.24	±	5.00	(5)
BRAIN	0.73	±	0.03	(5)	0.73	±	0.02	(5)	1.04	±	0.30	(5)	0.75	±	0.02	(5)
EYE (LEFT)	0.12	±	0.00	(5)	0.11	±	0.00	(5)	0.11	±	0.01	(5)	0.11	±	0.00	(5)
EYE (RIGHT)	0.11	±	0.01	(5)	0.11	±	0.00	(5)	0.11	±	0.00	(5)	0.11	±	0.00	(5)
SPLEEN	0.23	±	0.02	(5)	0.24	±	0.01	(5)	0.24	±	0.01	(5)	0.26	±	0.02	(5)
KIDNEY (LEFT)	0.40	±	0.01	(5)	0.39	±	0.01	(5)	0.40	±	0.01	(5)	0.42	±	0.01	(5)
KIDNEY (RIGHT)	0.40	±	0.00	(5)	0.39	±	0.00	(5)	0.40	±	0.01	(5)	0.41	±	0.01	(5)
THYMUS	0.21	±	0.01	(5)	0.23	±	0.02	(5)	0.22	±	0.01	(5)	0.22	±	0.01	(5)
TESTIS (LEFT)	0.47	±	0.02	(5)	0.50	±	0.03	(5)	0.52	±	0.02	(5)	0.51	±	0.01	(5)
TESTIS (RIGHT)	0.48	±	0.02	(5)	0.49	±	0.02	(5)	0.51	±	0.02	(5)	0.50	±	0.01	(5)
HEART	0.38	±	0.01	(5)	0.41	±	0.02	(5)	0.39	±	0.01	(5)	0.40	±	0.01	(5)
LIVER	3.77	±	0.13	(5)	3.83	±	0.17	(5)	3.64	±	0.12	(5)	3.51	±	0.09	(5)
LUNG (LEFT)	0.13	±	0.01	(5)	0.14	±	0.00	(5)	0.14	±	0.00	(5)	0.14	±	0.00	(5)
LUNG (RIGHT)	0.10	±	0.00	(5)	0.11	±	0.00	(5)	0.11	±	0.00	(5)	0.14	±	0.03	(5)
BRONCHUS	0.05	±	0.01	(5)	0.04	±	0.00	(5)	0.04	±	0.01	(5)	0.04	±	0.01	(5)

( ): number of animals

Table S3. Relative organs weights of male rats at 21 days after CNF exposure

Summary of Relative Organ Weights																
(mean ± S.E)						UNIT: g										SEX: MALE
Group:	Control				Low				Moderate				High			
BODY WEIGHT	377.98	±	14.17	(5)	376.66	±	9.16	(5)	374.10	±	4.15	(5)	391.46	±	9.88	(5)
BRAIN	0.55	±	0.02	(5)	0.055	±	0.01	(5)	0.57	±	0.01	(5)	0.53	±	0.02	(5)
EYE (LEFT)	0.10	±	0.00	(5)	0.10	±	0.00	(5)	0.10	±	0.00	(5)	0.09	±	0.01	(5)
EYE (RIGHT)	0.10	±	0.00	(5)	0.10	±	0.00	(5)	0.11	±	0.01	(5)	0.09	±	0.00	(5)
SPLEEN	0.20	±	0.02	(5)	0.20	±	0.03	(5)	0.18	±	0.01	(5)	0.20	±	0.01	(5)
KIDNEY (LEFT)	0.37	±	0.01	(5)	0.37	±	0.01	(5)	0.36	±	0.01	(5)	0.37	±	0.02	(5)
KIDNEY (RIGHT)	0.37	±	0.01	(5)	0.37	±	0.01	(5)	0.36	±	0.01	(5)	0.35	±	0.02	(5)
THYMUS	0.14	±	0.01	(5)	0.16	±	0.01	(5)	0.17	±	0.00	(5)	0.17	±	0.01	(5)
TESTIS (LEFT)	0.32	±	0.09	(5)	0.48	±	0.01*	(5)	0.46	±	0.01	(5)	0.45	±	0.01	(5)
TESTIS (RIGHT)	0.41	±	0.02	(5)	0.48	±	0.00	(5)	0.45	±	0.01	(5)	0.45	±	0.01	(5)
HEART	0.34	±	0.01	(5)	0.36	±	0.01	(5)	0.35	±	0.01	(5)	0.37	±	0.02	(5)
LIVER	3.05	±	0.05	(5)	3.09	±	0.07	(5)	2.98	±	0.09	(5)	2.88	±	0.05	(5)
LUNG (LEFT)	0.11	±	0.00	(5)	0.12	±	0.00	(5)	0.10	±	0.01	(5)	0.12	±	0.00	(5)
LUNG (RIGHT)	0.09	±	0.00	(5)	0.10	±	0.00	(5)	0.09	±	0.00	(5)	0.09	±	0.00	(5)
BRONCHUS	0.04	±	0.01	(5)	0.04	±	0.01	(5)	0.04	±	0.00	(5)	0.04	±	0.01	(5)

( ): number of animals, *p<0.05; comparison with control group

Table S4. Relative organs weights of female rats at 1 day after CNF exposure

Summary of Relative Organ Weights																
(mean ± S.E)						UNIT: g										SEX: FEMALE
Group:	Control				Low				Moderate				High			
BODY WEIGHT	179.06	±	3.59	(5)	180.66	±	1.95	(5)	185.92	±	2.14	(5)	180.74	±	1.89	(5)
BRAIN	1.06	±	0.02	(5)	1.02	±	0.01	(5)	1.01	±	0.02	(5)	1.07	±	0.02	(5)
EYE (LEFT)	0.16	±	0.01	(5)	0.14	±	0.00	(5)	0.15	±	0.01	(5)	0.16	±	0.00	(5)
EYE (RIGHT)	0.15	±	0.00	(5)	0.16	±	0.01	(5)	0.15	±	0.00	(5)	0.16	±	0.01	(5)
SPLEEN	0.23	±	0.01	(5)	0.22	±	0.01	(5)	0.24	±	0.01	(5)	0.24	±	0.02	(5)
KIDNEY (LEFT)	0.40	±	0.01	(5)	0.40	±	0.02	(5)	0.40	±	0.01	(5)	0.38	±	0.04	(5)
KIDNEY (RIGHT)	0.40	±	0.01	(5)	0.41	±	0.01	(5)	0.40	±	0.01	(5)	0.36	±	0.08	(5)
THYMUS	0.24	±	0.01	(5)	0.22	±	0.01	(5)	0.24	±	0.02	(5)	0.19	±	0.04	(5)
TESTIS (LEFT)	0.04	±	0.00	(5)	0.03	±	0.00	(5)	0.03	±	0.00	(5)	0.11	±	0.08	(5)
TESTIS (RIGHT)	0.03	±	0.00	(5)	0.03	±	0.00	(5)	0.03	±	0.00	(5)	0.11	±	0.09	(5)
HEART	0.40	±	0.01	(5)	0.42	±	0.01	(5)	0.39	±	0.01	(5)	0.42	±	0.00	(5)
LIVER	2.86	±	0.07	(5)	2.93	±	0.05	(5)	2.78	±	0.04	(5)	3.07	±	0.13	(5)
LUNG (LEFT)	0.17	±	0.00	(5)	0.16	±	0.00	(5)	0.18	±	0.00	(5)	0.17	±	0.01	(5)
LUNG (RIGHT)	0.13	±	0.01	(5)	0.13	±	0.00	(5)	0.13	±	0.00	(5)	0.15	±	0.01	(5)
BRONCHUS	0.06	±	0.00	(5)	0.07	±	0.01	(5)	0.06	±	0.01	(5)	0.06	±	0.01	(5)

( ): number of animals

Table S5. Hematology of male rats at 1 day after CNF exposure

Summary of Hematological Analysis																
Group: (mean ± S.E)	Control				Low				Moderate				High			
WBCB[1] (K/μL)	3.20	±	0.32	(5)	3.95	±	0.28	(5)	5.39	±	0.33*	(5)	4.77	±	0.34	(5)
RBC[2] (K/μL)	6.63	±	0.09	(5)	6.82	±	0.12	(5)	6.64	±	0.11	(5)	6.73	±	0.06	(5)
measHGB[3] (g/dL)	14.20	±	0.31	(5)	14.54	±	0.27	(5)	14.20	±	0.28	(5)	14.42	±	0.16	(5)
HCT[4] (%)	43.30	±	0.78	(5)	44.36	±	0.65	(5)	42.14	±	0.73	(5)	42.44	±	0.32	(5)
MCV[5] (fL)	65.46	±	0.42	(5)	65.02	±	0.97	(5)	63.50	±	0.99	(5)	63.20	±	1.00	(5)
MCH[6] (pg)	21.46	±	0.22	(5)	21.36	±	0.29	(5)	21.36	±	0.32	(5)	21.44	±	0.34	(5)
MCHC[7] (g/dL)	32.76	±	0.23	(5)	32.82	±	0.27	(5)	33.68	±	0.18*	(5)	33.96	±	0.23**	(5)
RDW[8] (%)	12.78	±	0.42	(5)	13.46	±	0.44	(5)	12.32	±	0.49	(5)	12.86	±	0.35	(5)
PLT[9] (%)	1296.80	±	70.21	(5)	1179.20	±	69.26	(5)	110.20	±	94.96	(5)	1038.20	±	91.93	(5)
MPV[10] (fL)	7.06	±	0.36	(5)	7.30	±	0.35	(5)	7.04	±	0.36	(5)	7.28	±	0.31	(5)
NEUT[11] (%)	15.58	±	5.64	(5)	25.14	±	5.37	(5)	17.10	±	5.56	(5)	12.32	±	5.58	(5)
LYM[12] (%)	81.10	±	5.99	(5)	70.58	±	5.62	(5)	79.00	±	6.00	(5)	84.38	±	6.04	(5)
MONO[13] (%)	1.50	±	0.44	(5)	2.38	±	0.53	(5)	2.16	±	0.66	(5)	1.64	±	0.66	(5)
EOS[14] (%)	1.24	±	0.17	(5)	1.16	±	0.16	(5)	0.94	±	0.15	(5)	0.92	±	0.15	(5)
LUC[15] (%)	0.60	±	0.09	(5)	0.72	±	0.09	(5)	0.76	±	0.10	(5)	0.74	±	0.12	(5)
BASO[16] (%)	0.02	±	0.02	(5)	0.02	±	0.02	(5)	0.04	±	0.02	(5)	0.02	±	0.02	(5)
abs_neuts[17] (g/dL)	0.48	±	0.18	(5)	0.93	±	0.14*	(5)	0.93	±	0.15*	(5)	0.55	±	0.14	(5)
abs_lymphs[18] (%)	2.60	±	0.38	(5)	2.85	±	0.39	(5)	4.25	±	0.46*	(5)	4.06	±	0.47	(5)
abs_monos[19] (K/μL)	0.05	±	0.01	(5)	0.09	±	0.02	(5)	0.11	±	0.03	(5)	0.08	±	0.03	(5)
abs_eos[20]	0.04	±	0.01	(5)	0.05	±	0.01	(5)	0.05	±	0.01	(5)	0.04	±	0.01	(5)
abs_lucs[21] (fL)	0.02	±	0.00	(5)	0.02	±	0.00	(5)	0.05	±	0.00*	(5)	0.04	±	0.00	(5)
abs_basos[22]	0.00	±	0.00	(5)	0.00	±	0.00	(5)	0.00	±	0.00	(5)	0.00	±	0.00	(5)
Retic[23]	5.32	±	0.28	(5)	5.11	±	0.16	(5)	4.66	±	0.16	(5)	4.49	±	0.14	(5)

( ): number of animals

1. White blood cell count using basophil method; 2. Red blood cells; 3. Hemoglobin; 4. Hematocrit; 5. Mean corpuscular volume; 6. Mean corpuscular hemoglobin; 7. Mean corpuscular hemoglobin concentration; 8. Red cell distribution width; 9. Platelets; 10. Mean platelet volume; 11. Percent of neutrophils; 12. Percent of lymphocytes; 13. Percent of monocytes; 14. Percent of eosinophils; 15. Percent of unstained cells; 16. Percent of basophils; 17. Absolute count of neutrophils; 18. Absolute count of lymphocytes; 19. Absolute count of monocytes; 20. Absolute count of eosinophils; 21. Absolute count of large unstained cells; 22. Absolute count of basophils; 23. Percent of reticulocytes. $^{*}p<0.05$ comparison with control group; $^{**}p<0.01$ comparison with control group

Table S6. Hematology of male rats at 3 days after CNF exposure

Summary of Hematological Analysis				
Group : (mean ± S.E)	Control	Low	Moderate	High
WBCB[1] (K/μL)	3.08 ± 0.67 (5)	4.69 ± 0.58 (5)	4.86 ± 0.49 (5)	4.97 ± 0.47 (5)
RBC[2] (K/μL)	6.52 ± 0.09 (5)	6.55 ± 0.08 (5)	6.63 ± 0.11 (5)	6.54 ± 0.10 (5)
measHGB[3] (g/dL)	13.66 ± 0.24 (5)	13.50 ± 0.11 (5)	13.60 ± 0.19 (5)	13.34 ± 0.16 (5)
HCT[4] (%)	43.54 ± 0.91 (5)	43.68 ± 0.51 (5)	43.68 ± 0.79 (5)	41.92 ± 0.49 (5)
MCV[5] (fL)	66.82 ± 0.96 (5)	66.62 ± 0.76 (5)	65.86 ± 0.46 (5)	64.16 ± 0.44* (5)
MCH[6] (pg)	20.96 ± 0.37 (5)	20.58 ± 0.33 (5)	20.52 ± 0.19 (5)	20.40 ± 0.17 (5)
MCHC[7] (g/dL)	31.36 ± 0.27 (5)	30.92 ± 0.27 (5)	31.14 ± 0.25 (5)	31.80 ± 0.10 (5)
RDW[8] (%)	12.22 ± 0.23 (5)	12.50 ± 0.24 (5)	12.98 ± 0.23* (5)	12.00 ± 0.20 (5)
PLT[9] (%)	1106.40 ± 27.95 (5)	1092.20 ± 23.99 (5)	1169.60 ± 23.31 (5)	1177.80 ± 26.64 (5)
MPV[10] (fL)	7.14 ± 0.09 (5)	7.12 ± 0.06 (5)	7.04 ± 0.06 (5)	6.58 ± 0.07** (5)
NEUT[11] (%)	19.44 ± 2.95 (5)	16.48 ± 1.98 (5)	17.84 ± 1.59 (5)	14.22 ± 1.49 (5)
LYM[12] (%)	77.14 ± 3.07 (5)	80.44 ± 2.01 (5)	79.06 ± 1.58 (5)	82.40 ± 1.37* (5)
MONO[13] (%)	1.52 ± 0.20 (5)	1.46 ± 0.14 (5)	1.60 ± 0.13 (5)	1.74 ± 0.08 (5)
EOS[14] (%)	1.22 ± 0.15 (5)	0.96 ± 0.17 (5)	0.80 ± 0.17 (5)	0.68 ± 0.20 (5)
LUC[15] (%)	0.66 ± 0.09 (5)	0.70 ± 0.10 (5)	0.68 ± 0.10 (5)	0.88 ± 0.09 (5)
BASO[16] (%)	0.02 ± 0.00 (5)	0.02 ± 0.02 (5)	0.02 ± 0.02 (5)	0.04 ± 0.02 (5)
abs_neuts[17] (g/dL)	0.59 ± 0.17 (5)	0.80 ± 0.17 (5)	0.90 ± 0.14 (5)	0.70 ± 0.13 (5)
abs_lymphs[18] (%)	2.39 ± 0.50 (5)	3.75 ± 0.39 (5)	3.80 ± 0.34 (5)	4.11 ± 0.34* (5)
abs_monos[19] (K/μL)	0.05 ± 0.01 (5)	0.07 ± 0.01 (5)	0.08 ± 0.01 (5)	0.08 ± 0.01 (5)
abs_eos[20]	0.04 ± 0.01 (5)	0.04 ± 0.00 (5)	0.04 ± 0.00 (5)	0.03 ± 0.01 (5)
abs_lucs[21] (fL)	0.02 ± 0.01 (5)	0.04 ± 0.01 (5)	0.03 ± 0.01 (5)	0.05 ± 0.01 (5)
abs_basos[22]	0.00 ± 0.00 (5)	0.00 ± 0.00 (5)	0.00 ± 0.00 (5)	0.00 ± 0.00 (5)
Retic[23]	4.43 ± 0.38 (5)	4.48 ± 0.37 (5)	4.18 ± 0.35 (5)	4.30 ± 0.33 (5)

( ): number of animals

1. White blood cell count using basophil method; 2. Red blood cells; 3. Hemoglobin; 4. Hematocrit; 5. Mean corpuscular volume; 6. Mean corpuscular hemoglobin; 7. Mean corpuscular hemoglobin concentration; 8. Red cell distribution width; 9. Platelets; 10. Mean platelet volume; 11. Percent of neutrophils; 12. Percent of lymphocytes; 13. Percent of monocytes; 14. Percent of eosinophils; 15. Percent of unstained cells; 16. Percent of basophils; 17. Absolute count of neutrophils; 18. Absolute count of lymphocytes; 19. Absolute count of monocytes; 20. Absolute count of eosinophils; 21. Absolute count of large unstained cells; 22. Absolute count of basophils; 23. Percent of reticulocytes. $^{*}p$<0.05 comparison with control group; $^{**}p$<0.01 comparison with control group

Table S7. Hematology of male rats at 21 days after CNF exposure

Summary of Hematological Analysis				
Group: (mean ± S.E)	Control	Low	Moderate	High
WBCB[1] (K/μL)	3.19 ± 0.31 (5)	3.62 ± 0.40 (5)	4.52 ± 0.43 (5)	4.25 ± 0.88 (5)
RBC[2] (K/μL)	7.49 ± 0.09 (5)	7.71 ± 0.08 (5)	7.82 ± 0.15 (5)	7.31 ± 0.12 (5)
measHGB[3] (g/dL)	14.70 ± 0.31 (5)	15.04 ± 0.26 (5)	15.06 ± 0.22 (5)	14.28 ± 0.22 (5)
HCT[4] (%)	45.44 ± 0.74 (5)	46.98 ± 0.68 (5)	46.24 ± 0.75 (5)	43.56 ± 0.70 (5)
MCV[5] (fL)	60.70 ± 0.47 (5)	60.90 ± 0.47 (5)	59.20 ± 0.60 (5)	59.60 ± 0.55 (5)
MCH[6] (pg)	19.62 ± 0.30 (5)	19.52 ± 0.31 (5)	19.30 ± 0.24 (5)	19.54 ± 0.17 (5)
MCHC[7] (g/dL)	32.34 ± 0.27 (5)	32.06 ± 0.30 (5)	32.60 ± 0.20 (5)	32.80 ± 0.18 (5)
RDW[8] (%)	10.92 ± 0.22 (5)	11.18 ± 0.23 (5)	11.04 ± 0.25 (5)	11.18 ± 0.17 (5)
PLT[9] (%)	1159.20 ± 53.33 (5)	1178.00 ± 49.22 (5)	1184.60 ± 64.08 (5)	1203.40 ± 63.90 (5)
MPV[10] (fL)	7.06 ± 0.10 (5)	7.46 ± 0.08 (5)	7.06 ± 0.13 (5)	6.98 ± 0.16 (5)
NEUT[11] (%)	22.14 ± 1.96 (5)	16.94 ± 2.46 (5)	14.22 ± 2.58 (5)	18.98 ± 1.46 (5)
LYM[12] (%)	74.04 ± 2.24 (5)	79.54 ± 2.83 (5)	82.60 ± 2.88 (5)	76.58 ± 1.64 (5)
MONO[13] (%)	1.32 ± 0.32 (5)	1.14 ± 0.32 (5)	1.08 ± 0.29 (5)	1.68 ± 0.27 (5)
EOS[14] (%)	2.16 ± 0.62 (5)	1.80 ± 0.65 (5)	1.64 ± 0.60 (5)	2.20 ± 0.43 (5)
LUC[15] (%)	0.30 ± 0.07 (5)	0.52 ± 0.07 (5)	0.46 ± 0.07 (5)	0.52 ± 0.04 (5)
BASO[16] (%)	0.00 ± 0.02 (5)	0.06 ± 0.02* (5)	0.00 ± 0.02 (5)	0.02 ± 0.02 (5)
abs_neuts[17] (g/dL)	0.68 ± 0.10 (5)	0.61 ± 0.11 (5)	0.65 ± 0.12 (5)	0.81 ± 0.11 (5)
abs_lymphs[18] (%)	2.39 ± 0.23 (5)	2.88 ± 0.37 (5)	3.74 ± 0.38 (5)	3.25 ± 0.78 (5)
abs_monos[19] (K/μL)	0.04 ± 0.01 (5)	0.04 ± 0.01 (5)	0.05 ± 0.01 (5)	0.07 ± 0.01 (5)
abs_eos[20]	0.07 ± 0.02 (5)	0.06 ± 0.02 (5)	0.06 ± 0.02 (5)	0.09 ± 0.01 (5)
abs_lucs[21] (fL)	0.01 ± 0.00 (5)	0.02 ± 0.00 (5)	0.02 ± 0.00 (5)	0.02 ± 0.00 (5)
abs_basos[22]	0.00 ± 0.00 (5)	0.00 ± 0.00 (5)	0.00 ± 0.00 (5)	0.00 ± 0.00 (5)
Retic[23]	2.25 ± 0.72 (5)	2.85 ± 0.72 (5)	2.17 ± 0.76 (5)	2.53 ± 0.17 (5)

( ): number of animals

1. White blood cell count using basophil method; 2. Red blood cells; 3. Hemoglobin; 4. Hematocrit; 5. Mean corpuscular volume; 6. Mean corpuscular hemoglobin; 7. Mean corpuscular hemoglobin concentration; 8. Red cell distribution width; 9. Platelets; 10. Mean platelet volume; 11. Percent of neutrophils; 12. Percent of lymphocytes; 13. Percent of monocytes; 14. Percent of eosinophils; 15. Percent of unstained cells; 16. Percent of basophils; 17. Absolute count of neutrophils; 18. Absolute count of lymphocytes; 19. Absolute count of monocytes; 20. Absolute count of eosinophils; 21. Absolute count of large unstained cells; 22. Absolute count of basophils; 23. Percent of reticulocytes. $^{*}p<0.05$ comparison with control group

Table S8. Hematology of female rats at 1 day after CNF exposure

Summary of Hematological Analysis				
Group:<br>(mean ± S.E)	Control	Low	Moderate	High
WBCB[1] (K/µL)	2.94 ± 0.24 (5)	4.72 ± 0.46* (4)	3.97 ± 0.76 (4)	4.69 ± 0.63* (4)
RBC[2] (K/µL)	7.31 ± 0.12 (5)	7.48 ± 0.13 (4)	7.39 ± 0.13 (4)	7.43 ± 0.12 (4)
measHGB[3] (g/dL)	15.16 ± 0.21 (5)	15.53 ± 0.21 (4)	15.93 ± 0.20 (4)	14.93 ± 0.17 (4)
HCT[4] (%)	45.48 ± 1.07 (5)	44.43 ± 1.04 (4)	45.40 ± 0.67 (4)	43.13 ± 0.38 (4)
MCV[5] (fL)	62.22 ± 1.21 (5)	59.45 ± 1.19 (4)	61.45 ± 0.36 (4)	58.05 ± 0.57* (4)
MCH[6] (pg)	20.76 ± 0.16 (5)	20.75 ± 0.14 (4)	21.58 ± 0.11 (4)	20.10 ± 0.11 (4)
MCHC[7] (g/dL)	33.42 ± 0.44 (5)	34.90 ± 0.44* (4)	35.15 ± 0.22* (4)	34.65 ± 0.23 (4)
RDW[8] (%)	11.08 ± 0.29 (5)	10.80 ± 0.21 (4)	11.20 ± 0.15 (4)	10.40 ± 0.14 (4)
PLT[9] (%)	1008.00 ± 116.87 (5)	1194.75 ± 119.84 (4)	971.00 ± 131.52 (4)	1168.25 ± 124.30 (4)
MPV[10] (fL)	7.46 ± 0.19 (5)	6.95 ± 0.18 (4)	7.35 ± 0.21 (4)	7.20 ± 0.25 (4)
NEUT[11] (%)	24.06 ± 6.73 (5)	14.20 ± 4.75 (4)	16.35 ± 4.22 (4)	9.08 ± 2.40 (4)
LYM[12] (%)	69.26 ± 9.58 (5)	83.40 ± 4.90* (4)	80.38 ± 4.40 (4)	88.00 ± 2.38* (4)
MONO[13] (%)	0.98 ± 0.16 (5)	1.00 ± 0.10 (4)	1.75 ± 0.08 (4)	1.43 ± 0.04 (4)
EOS[14] (%)	5.14 ± 3.70 (5)	0.93 ± 0.28 (4)	0.75 ± 0.28 (4)	0.90 ± 0.16 (4)
LUC[15] (%)	0.54 ± 0.17 (5)	0.43 ± 0.16 (4)	0.73 ± 0.10 (4)	0.58 ± 0.08 (4)
BASO[16] (%)	0.06 ± 0.02 (5)	0.05 ± 0.02 (4)	0.05 ± 0.02 (4)	0.05 ± 0.02 (4)
abs_neuts[17] (g/dL)	0.68 ± 0.19 (5)	0.66 ± 0.14 (4)	0.64 ± 0.10 (4)	0.42 ± 0.10 (4)
abs_lymphs[18] (%)	2.07 ± 0.38 (5)	3.94 ± 0.42 (4)	3.21 ± 0.73 (4)	4.13 ± 0.58* (4)
abs_monos[19] (K/µL)	0.03 ± 0.00 (5)	0.05 ± 0.00 (4)	0.07 ± 0.01 (4)	0.07 ± 0.01 (4)
abs_eos[20]	0.15 ± 0.11 (5)	0.04 ± 0.01 (4)	0.03 ± 0.01 (4)	0.04 ± 0.01 (4)
abs_lucs[21] (fL)	0.02 ± 0.01 (5)	0.02 ± 0.01 (4)	0.03 ± 0.01 (4)	0.03 ± 0.01 (4)
abs_basos[22]	0.00 ± 0.00 (5)	0.00 ± 0.00 (4)	0.00 ± 0.00 (4)	0.00 ± 0.00 (4)
Retic[23]	1.85 ± 0.25 (5)	2.02 ± 0.23 (4)	1.86 ± 0.25 (4)	1.58 ± 0.25 (4)

( ): number of animals

1. White blood cell count using basophil method; 2. Red blood cells; 3. Hemoglobin; 4. Hematocrit; 5. Mean corpuscular volume; 6. Mean corpuscular hemoglobin; 7. Mean corpuscular hemoglobin concentration; 8. Red cell distribution width; 9. Platelets; 10. Mean platelet volume; 11. Percent of neutrophils; 12. Percent of lymphocytes; 13. Percent of monocytes; 14. Percent of eosinophils; 15. Percent of unstained cells; 16. Percent of basophils; 17. Absolute count of neutrophils; 18. Absolute count of lymphocytes; 19. Absolute count of monocytes; 20. Absolute count of eosinophils; 21. Absolute count of large unstained cells; 22. Absolute count of basophils; 23. Percent of reticulocytes.

$^{*}p<0.05$ comparison with control group; $^{**}p<0.01$ comparison with control group

Table S9. Serum biochemical test of male rats at 1 day after CNF exposure

Summary of Serum Biochemical Analysis																
Group: (mean ± S.E)	Control				Low				Moderate				High			
ALB[1] (g/dL)	2.38	±	0.04	(5)	2.30	±	0.11	(5)	2.32	±	0.10	(5)	2.32	±	0.06	(5)
ALP[2] (IU/L)	652.40	±	55.31	(5)	675.80	±	33.45	(5)	703.00	±	66.95	(5)	635.20	±	77.60	(5)
CA[3] (mg/dL)	8.62	±	0.28	(5)	8.58	±	0.29	(5)	8.32	±	0.33	(5)	8.40	±	0.13	(5)
CHO[4] (mg/dL)	72.60	±	4.02	(5)	70.80	±	5.05	(5)	77.40	±	8.20	(5)	68.00	±	5.36	(5)
CRE[5] (mg/dL)	0.48	±	0.02	(5)	0.50	±	0.02	(5)	0.44	±	0.04	(5)	0.43	±	0.02	(5)
γ-GT[6] (IU/L)	0.60	±	0.24	(5)	0.60	±	0.24	(5)	0.80	±	0.20	(5)	0.60	±	0.24	(5)
GLU[7]	73.60	±	2.94	(5)	66.80	±	4.33	(5)	81.20	±	8.13	(5)	71.20	±	4.89	(5)
GOT[8] (mg/dL)	228.00	±	25.06	(5)	204.20	±	23.06	(5)	215.40	±	10.96	(5)	183.20	±	11.56	(5)
ALT[9] (IU/L)	34.60	±	3.50	(5)	32.00	±	2.17	(5)	36.20	±	4.73	(5)	33.00	±	0.63	(5)
IP[10] (mg/dL)	11.00	±	0.33	(5)	10.30	±	0.39	(5)	9.52	±	0.24**	(5)	9.22	±	0.19**	(5)
LDH[11] (IU/L)	3048.60	±	334.83	(5)	2672.00	±	391.15	(5)	2973.60	±	274.11	(5)	2224.60	±	249.28	(5)
MG[12] (mg/dL)	3.16	±	0.15	(5)	3.02	±	0.16	(5)	2.66	±	0.16	(5)	2.44	±	0.07**	(5)
TP[13] (g/dL)	5.46	±	0.14	(5)	5.28	±	0.18	(5)	5.30	±	0.23	(5)	5.26	±	0.09	(5)
UA[13] (mg/dL)	1.64	±	0.23	(5)	1.76	±	0.21	(5)	1.22	±	0.31	(5)	1.40	±	0.07	(5)
BUN[15] (mg/dL)	11.68	±	1.03	(5)	12.42	±	0.56	(5)	12.50	±	1.00	(5)	11.88	±	0.53	(5)
TBIL[16] (mg/dL)	0.01	±	0.00	(5)	0.01	±	0.00	(5)	0.02	±	0.01	(5)	0.02	±	0.00	(5)
TG[17] (mg/dL)	47.00	±	10.63	(5)	51.60	±	3.49	(5)	62.00	±	7.57	(5)	52.80	±	6.46	(5)
CK[18] (IU/L)	998.20	±	118.01	(5)	1265.80	±	385.58	(5)	837.60	±	33.20	(5)	835.80	±	107.40	(5)
Na[19] (mmol/L)	172.60	±	9.00	(5)	162.40	±	4.77	(5)	153.40	±	3.91	(5)	148.40	±	0.51*	(5)
K[20] (mmol/L)	5.38	±	0.39	(5)	5.16	±	0.29	(5)	4.84	±	0.13	(5)	4.60	±	0.11	(5)
Cl[21] (mmol/L)	102.00	±	6.69	(5)	90.60	±	5.56	(5)	88.00	±	3.63	(5)	96.20	±	1.56	(5)

( ): number of animals.; 1. Albumin; 2. Alkaline phosphatase; 3. Calcium; 4. Cholesterol; 5. Creatinine; 6. Gamma glutamyl transpeptidase; 7. Glucose; 8. Glutamic oxaloacetic transaminase; 9. Glutamic pyruvic transaminase; 10. Inorganic phosphorus; 11. Lactate Dehydrogenase; 12. Magnesium; 13. Total protein; 14. Uric acid; 15. Blood urea nitrogen; 16. Total bilirubin; 17. Triglyceride; 18. Creatine Kinase; 19. Sodium; 20. Potassium; 21. Chloride.

$^{*}p<0.05$ comparison with control group; $^{**}p<0.01$ comparison with control group

Table S10. Serum biochemical test of male rats at 3 days after CNF exposure

Summary of Serum Biochemical Analysis				
Group : (mean ± S.E)	Control	Low	Moderate	High
ALB[1] (g/dL)	2.16 ± 0.07 (5)	1.98 ± 0.08 (5)	2.10 ± 0.06 (5)	2.06 ± 0.02 (5)
ALP[2] (IU/L)	733.00 ± 16.31 (5)	806.20 ± 91.77 (5)	674.20 ± 74.10 (5)	654.60 ± 60.31 (5)
CA[3] (mg/dL)	8.18 ± 0.36 (5)	7.74 ± 0.25 (5)	8.08 ± 0.14 (5)	7.80 ± 0.11 (5)
CHO[4] (mg/dL)	76.40 ± 5.70 (5)	68.60 ± 7.50 (5)	75.20 ± 3.40 (5)	71.80 ± 3.87 (5)
CRE[5] (mg/dL)	0.48 ± 0.00 (5)	0.44 ± 0.01 (5)	0.42 ± 0.01* (5)	0.40 ± 0.01** (5)
γ-GT[6] (IU/L)	0.60 ± 0.24 (5)	0.80 ± 0.20 (5)	0.80 ± 0.20 (5)	0.60 ± 0.24 (5)
GLU[7]	153.00 ± 11.55 (5)	163.80 ± 6.37 (5)	147.40 ± 8.33 (5)	169.40 ± 9.93 (5)
GOT[8] (mg/dL)	187.60 ± 15.87 (5)	140.60 ± 6.19 (5)	152.40 ± 9.18 (5)	115.60 ± 4.77* (5)
ALT[9] (IU/L)	42.80 ± 4.13 (5)	34.80 ± 2.06 (5)	38.80 ± 1.85 (5)	35.20 ± 2.71 (5)
IP[10] (mg/dL)	10.16 ± 0.39 (5)	9.50 ± 0.22 (5)	9.32 ± 0.24 (5)	8.44 ± 0.15** (5)
LDH[11] (IU/L)	2465.60 ± 262.60 (5)	1731.40 ± 112.80* (5)	1913.00 ± 104.82 (5)	1149.40 ± 166.50** (5)
MG[12] (mg/dL)	2.80 ± 0.04 (5)	2.38 ± 0.08** (5)	2.48 ± 0.09* (5)	2.26 ± 0.05** (5)
TP[13] (g/dL)	5.12 ± 0.17 (5)	4.70 ± 0.16 (5)	4.92 ± 0.22 (5)	4.76 ± 0.10 (5)
UA[13] (mg/dL)	1.36 ± 0.11 (5)	1.06 ± 0.08 (5)	1.16 ± 0.02 (5)	1.08 ± 0.09 (5)
BUN[15] (mg/dL)	14.36 ± 1.54 (5)	16.86 ± 0.29 (5)	14.76 ± 0.84 (5)	12.42 ± 0.55 (5)
TBIL[16] (mg/dL)	0.01 ± 0.00 (5)	0.02 ± 0.01 (5)	0.02 ± 0.01 (5)	0.03 ± 0.01 (5)
TG[17] (mg/dL)	55.20 ± 8.48 (5)	64.80 ± 10.73 (5)	63.40 ± 7.55 (5)	81.60 ± 5.64 (5)
CK[18] (IU/L)	1498.00 ± 150.05 (5)	1081.60 ± 81.91 (5)	1415.60 ± 206.92 (5)	663.00 ± 41.14** (5)
Na[19] (mmol/L)	160.40 ± 3.06 (5)	148.80 ± 1.74* (5)	146.20 ± 3.53** (5)	139.20 ± 1.24** (5)
K[20] (mmol/L)	4.78 ± 0.08 (5)	4.58 ± 0.14 (5)	4.56 ± 0.17 (5)	4.16 ± 0.05** (5)
Cl[21] (mmol/L)	88.00 ± 3.05 (5)	82.00 ± 2.17 (5)	83.80 ± 4.16 (5)	86.80 ± 1.46 (5)

( ): number of animals; 1. Albumin; 2. Alkaline phosphatase; 3. Calcium; 4. Cholesterol; 5. Creatinine; 6. Gamma glutamyl transpeptidase; 7. Glucose; 8. Glutamic oxaloacetic transaminase; 9. Glutamic pyruvic transaminase; 10. Inorganic phosphorus; 11. Lactate Dehydrogenase; 12. Magnesium; 13. Total protein; 14. Uric acid; 15. Blood urea nitrogen; 16. Total bilirubin; 17. Triglyceride; 18. Creatine Kinase; 19. Sodium; 20. Potassium; 21. Chloride.
$^{*}p<0.05$ comparison with control group; $^{**}p<0.01$ comparison with control group

Table S11. Serum biochemical test of male rats at 21 days after CNF exposure

Summary of Serum Biochemical Analysis				
Group: (mean ± S.E)	Control	Low	Moderate	High
ALB[1] (g/dL)	2.16 ± 0.02 (5)	2.28 ± 0.05 (5)	2.30 ± 0.03 (5)	2.20 ± 0.04 (5)
ALP[2] (IU/L)	458.80 ± 29.86 (5)	525.00 ± 72.24 (5)	502.60 ± 45.04 (5)	483.00 ± 39.82 (5)
CA[3] (mg/dL)	9.36 ± 0.04 (5)	9.06 ± 0.16 (5)	9.18 ± 0.15 (5)	9.26 ± 0.07 (5)
CHO[4] (mg/dL)	64.40 ± 4.72 (5)	72.00 ± 6.00 (5)	59.80 ± 1.98 (5)	64.60 ± 7.32 (5)
CRE[5] (mg/dL)	0.53 ± 0.03 (5)	0.50 ± 0.04 (5)	0.60 ± 0.02 (5)	0.45 ± 0.02 (5)
γ-GT[6] (IU/L)	0.20 ± 0.20 (5)	0.00 ± 0.00 (5)	0.00 ± 0.00 (5)	0.20 ± 0.20 (5)
GLU[7]	121.40 ± 4.98 (5)	131.40 ± 5.63 (5)	146.00 ± 11.82 (5)	148.40 ± 7.45 (5)
GOT[8] (mg/dL)	194.00 ± 7.09 (5)	164.60 ± 8.52 (5)	183.40 ± 6.57 (5)	111.00 ± 10.20** (5)
ALT[9] (IU/L)	42.60 ± 2.32 (5)	39.00 ± 2.77 (5)	38.20 ± 2.52 (5)	36.80 ± 2.60 (5)
IP[10] (mg/dL)	9.66 ± 0.22 (5)	8.54 ± 0.17** (5)	8.92 ± 0.15* (5)	8.34 ± 0.14** (5)
LDH[11] (IU/L)	2735.40 ± 175.66 (5)	2233.00 ± 157.38 (5)	2654.60 ± 170.32 (5)	1264.60 ± 233.63** (5)
MG[12] (mg/dL)	2.98 ± 0.09 (5)	2.86 ± 0.05 (5)	2.72 ± 0.07* (5)	2.62 ± 0.06** (5)
TP[13] (g/dL)	5.82 ± 0.09 (5)	5.72 ± 0.05 (5)	5.92 ± 0.07 (5)	5.70 ± 0.10 (5)
UA[13] (mg/dL)	1.48 ± 0.08 (5)	1.44 ± 0.12 (5)	1.74 ± 0.16 (5)	1.16 ± 0.04 (5)
BUN[15] (mg/dL)	18.50 ± 0.44 (5)	17.78 ± 0.43 (5)	21.42 ± 0.79* (5)	16.22 ± 1.04 (5)
TBIL[16] (mg/dL)	0.03 ± 0.01 (5)	0.05 ± 0.02 (5)	0.03 ± 0.00 (5)	0.05 ± 0.00 (5)
TG[17] (mg/dL)	46.20 ± 5.62 (5)	61.80 ± 8.56 (5)	71.60 ± 10.13 (5)	79.80 ± 16.23 (5)
CK[18] (IU/L)	2827.60 ± 667.43 (5)	2311.00 ± 497.66 (5)	3478.40 ± 1196.73 (5)	988.20 ± 100.35 (5)
Na[19] (mmol/L)	144.00 ± 1.58 (5)	140.20 ± 2.48 (5)	135.00 ± 0.71** (5)	136.60 ± 0.68* (5)
K[20] (mmol/L)	4.76 ± 0.05 (5)	4.32 ± 0.20* (5)	4.62 ± 0.07 (5)	4.30 ± 0.05* (5)
Cl[21] (mmol/L)	102.40 ± 0.93 (5)	101.40 ± 1.60 (5)	99.20 ± 0.49 (5)	102.20 ± 0.37 (5)

( ): number of animals; 1. Albumin; 2. Alkaline phosphatase; 3. Calcium; 4. Cholesterol; 5. Creatinine; 6. Gamma glutamyl transpeptidase; 7. Glucose; 8. Glutamic oxaloacetic transaminase; 9. Glutamic pyruvic transaminase; 10. Inorganic phosphorus; 11. Lactate Dehydrogenase; 12. Magnesium; 13. Total protein; 14. Uric acid; 15. Blood urea nitrogen; 16. Total bilirubin; 17. Triglyceride; 18. Creatine Kinase; 19. Sodium; 20. Potassium; 21. Chloride.

$^{*}p<0.05$ comparison with control group; $^{**}p<0.01$ comparison with control group

Table S12. Serum biochemical test of female rats at 1 day after CNF exposure

Summary of Serum Biochemical Analysis				
Group: (mean ± S.E)	Control	Low	Moderate	High
ALB[1] (g/dL)	2.50 ± 0.09 (5)	2.80 ± 0.07 (4)	2.68 ± 0.12 (5)	2.72 ± 0.13 (5)
ALP[2] (IU/L)	418.20 ± 26.98 (5)	423.75 ± 58.07 (4)	396.00 ± 46.48 (5)	333.00 ± 31.01 (5)
CA[3] (mg/dL)	9.36 ± 0.17 (5)	9.95 ± 0.03 (4)	10.22 ± 0.21 (5)	9.78 ± 0.43 (5)
CHO[4] (mg/dL)	89.40 ± 5.41 (5)	101.25 ± 4.70 (4)	92.00 ± 8.44 (5)	93.60 ± 10.04 (5)
CRE[5] (mg/dL)	0.60 ± 0.04 (5)	0.60 ± 0.04 (4)	0.53 ± 0.03 (5)	0.53 ± 0.03 (5)
γ-GT[6] (IU/L)	0.80 ± 0.20 (5)	1.00 ± 0.00 (4)	0.80 ± 0.20 (5)	1.00 ± 0.00 (5)
GLU[7]	77.20 ± 5.83 (5)	102.50 ± 12.26 (4)	74.60 ± 6.05 (5)	108.00 ± 10.21 (5)
GOT[8] (mg/dL)	239.20 ± 7.91 (5)	245.75 ± 13.37 (4)	254.40 ± 32.80 (5)	204.40 ± 19.81 (5)
ALT[9] (IU/L)	36.60 ± 1.89 (5)	43.50 ± 2.14 (4)	41.20 ± 4.37 (5)	34.60 ± 2.87 (5)
IP[10] (mg/dL)	10.58 ± 0.37 (5)	10.10 ± 0.30 (4)	9.38 ± 0.20* (5)	8.62 ± 0.12** (5)
LDH[11] (IU/L)	3420.80 ± 176.18 (5)	3600.00 ± 46.19 (4)	3500.80 ± 522.14 (5)	2751.20 ± 359.61 (5)
MG[12] (mg/dL)	3.58 ± 0.10 (5)	3.60 ± 0.23 (4)	3.24 ± 0.15 (5)	3.12 ± 0.14 (5)
TP[13] (g/dL)	5.88 ± 0.18 (5)	6.40 ± 0.08 (4)	6.26 ± 0.24 (5)	6.26 ± 0.28 (5)
UA[13] (mg/dL)	1.40 ± 0.08 (5)	1.70 ± 0.10 (4)	1.62 ± 0.12 (5)	1.52 ± 0.12 (5)
BUN[15] (mg/dL)	20.50 ± 0.60 (5)	20.50 ± 0.64 (4)	15.62 ± 1.90* (5)	18.38 ± 1.28 (5)
TBIL[16] (mg/dL)	0.02 ± 0.00 (5)	0.02 ± 0.00 (4)	0.01 ± 0.01 (5)	0.02 ± 0.01 (5)
TG[17] (mg/dL)	25.20 ± 2.20 (5)	47.25 ± 4.57 (4)	59.25 ± 5.22 (5)	56.60 ± 8.65* (5)
CK[18] (IU/L)	1265.40 ± 104.46 (5)	1239.00 ± 69.77 (4)	1060.00 ± 195.36 (5)	905.00 ± 143.11 (5)
Na[19] (mmol/L)	177.60 ± 4.58 (5)	178.67 ± 5.03 (4)	170.75 ± 6.61 (5)	164.40 ± 6.71 (5)
K[20] (mmol/L)	4.84 ± 0.14 (5)	5.27 ± 0.28 (4)	4.95 ± 0.17 (5)	5.02 ± 0.23 (5)
Cl[21] (mmol/L)	102.80 ± 4.42 (5)	110.00 ± 2.32 (4)	111.75 ± 4.84 (5)	116.60 ± 8.03 (5)

( ): number of animals; 1. Albumin; 2. Alkaline phosphatase; 3. Calcium; 4. Cholesterol; 5. Creatinine; 6. Gamma glutamyl transpeptidase; 7. Glucose; 8. Glutamic oxaloacetic transaminase; 9. Glutamic pyruvic transaminase; 10. Inorganic phosphorus; 11. Lactate Dehydrogenase; 12. Magnesium; 13. Total protein; 14. Uric acid; 15. Blood urea nitrogen; 16. Total bilirubin; 17. Triglyceride; 18. Creatine Kinase; 19. Sodium; 20. Potassium; 21. Chloride.

*p<0.05 comparison with control group; **p<0.01 comparison with control group
